# Content determination of ampicillin by Ni(ii)-mediated UV-Vis spectrophotometry

**DOI:** 10.1039/d2ra00116k

**Published:** 2022-03-29

**Authors:** Yu Lin, Siyuan Cen

**Affiliations:** Faculty of Pharmacy, Guangxi University of Chinese Medicine Nanning 530001 People's Republic of China wzgzly@126.com; Faculty of International Education, Guangxi University of Chinese Medicine Nanning 530001 People's Republic of China

## Abstract

Ampicillin could be degraded under alkaline conditions, of which the degradation products formed a complex with Ni^2+^ in a ratio of 2 : 1 in ammonium hydroxide. According to the study, it was found that there was a characteristic absorption peak at the wavelength of 269 nm, and the molar absorption coefficient and the stability constant of the complex was 4.28 × 10^3^ L mol^−1^ cm^−1^ and 5.95 × 10^9^, respectively. The linear relationship between the concentration and absorbance was favorable at the range of 17.47–69.88 μg mL^−1^. The regression equation was calculated as *A* = 0.0124*C* + 0.0053. The *R*^2^ was 0.9990 and the detection limit was 0.52 μg mL^−1^. Thus, the Ni^2+^ complex-based ultraviolet spectrophotometry has been created as a new method for indirect determination of ampicillin, with recovery rates from 98.68 to 102.7%, and the relative standard deviation (RSD) is from 0.7% to 1.7%, when applied for determining the content of practical samples.

## Abstract 2

Ampicillin could be degraded under alkaline conditions, of which the degradation products formed a complex with Ni^2+^ in a ratio of 2 : 1 in ammonium hydroxide.

## Graphical abstract

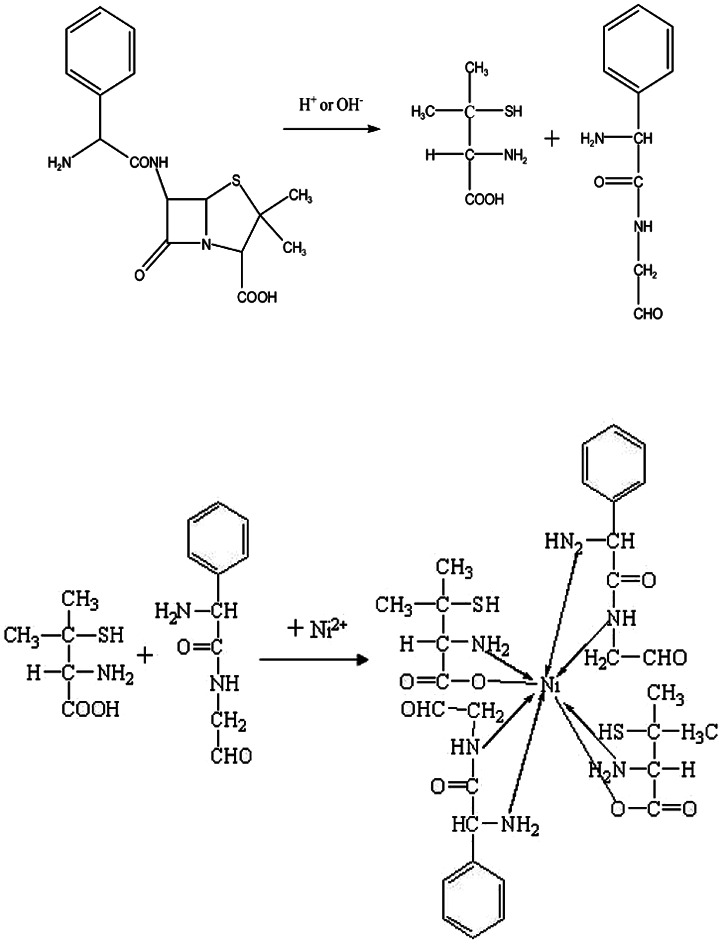

## 1. Introduction

Ampicillin, a semisynthetic penicillin, is mainly applied for the sensitive bacteria-induced infection of the urinary system, respiratory system, biliary tract and intestinal tract, as well as endocarditis and cephalomeningitis. Due to its wide antibacterial spectrum, oral administration and convenient use, it has been widely used. However, penicillin is widely used in livestock and poultry breeding, resulting in its residues in livestock products. Long-term consumption of food containing penicillin residues can cause bacterial resistance, resulting in imbalance of the human microbial environment. People who are allergic to antibiotics can also have allergic reactions, leading to immune anemia, rashes, *etc.* The Food Law of the World Food and Agriculture Organization and the World Health Organization has clear regulations on the maximum residue limit of antibiotics in food. The European Union and the US Food and Drug Administration also have specific and strict regulations, expressly prohibiting the marketing of milk with antibiotic residues exceeding the maximum residue limit. Many countries also explicitly list ampicillin as a mandatory inspection item for imported food.

At present, the methods for determining the ampicillin include liquid chromatography,^1–6^ molecularly imprinted solid phase extraction,^7,8^ spectrophotometry,^6–13^ electrochemical method,^14–18^ fluorescence method,^19–21^ circular dichroism spectroscopy,^22^*etc.* Due to its simple equipment, convenient operation, low price, and easy popularization, ultraviolet visible spectrophotometry has a wide range of applications in drug analysis. However, for most pharmaceutical preparations, other co-existing components (including solvents and excipients) have different degrees of interference to the determination of main components, so the application of direct UV-Vis spectrophotometry in pharmaceutical analysis is still limited. Since the characteristic absorption of ampicillin is at 202 nm, there is more interference near the characteristic wavelength. In addition, ampicillin is insoluble in water, but soluble in dilute alkali or acid, and is easily degraded in alkali or acid to form penicillamine and benzyl-containing penicillaldehyde. Therefore, indirect UV-Vis spectrophotometry is considered for the determination of ampicillin, which can not only eliminate the interference of coexisting components, but also improve the selectivity of the method.

In this work, the study discovered that Ni^2+^ could form the complex with degradation products of ampicillin under the alkaline condition, which had a characteristic absorption peak at the wavelength of 269 nm. Besides, it was found that the absorbance of the complex was enhanced with the increasing of ampicillin concentration so as to find out a new method for the determination of ampicillin content based on Ni^2+^ complex.

## 2. Experiments

### 2.1 Experimental apparatus, drugs and reagents

UV-2100 UV-Vis spectrophotometer (Beijing Beifen Ruili Co., Ltd, China) and Nexus-470 Fourier infrared spectrometric analyzer (Thermo Nicolet Corporation Co., Ltd, USA) were utilized for the determination of ampicillin. Ampicillin standard substance were purchased from China National Institute for Drug and Biological Products Control.

### 2.2 Preparation of stock solution

#### 2.2.1 Degradation solution of ampicillin

The mixtures of 0.4070 g ampicillin standard substances and 5.50 g NaOH were weighed accurately and dissolved in the distilled water and then heated in the boiling water bath for 2 h. Appropriate amounts of 1 mol L^−1^ HCl solution were dripped into the mixture to neutralize excess NaOH. Then the mixture was placed in a 100 mL volumetric flask after refrigeration and shaken evenly, so as to obtain the degradation solution of 1.00 × 10^−2^ mol L^−1^ which would be diluted for 10 times by distilled water.

#### 2.2.2 Ni^2+^ solution

0.1467 g of nickel powders were weighed accurately and dissolved by certain amounts of 12mol L^−1^ HCl, which was placed in a 250 mL volumetric flask in order to obtain the Ni^2+^ solution of 1.00 × 10^−2^ mol L^−1^ which would be diluted for 10 times by distilled water.

### 2.3 Experimental methods

1.00 × 10^−3^ mol L^−1^ degradation solution of ampicillin, 0.20 mL 1% ammonium hydroxide and 1.00 × 10^−3^ mol L^−1^ Ni^2+^ solution were added into a 10 mL volumetric flask in order, which was diluted with distilled water to volume. After the reaction for 1 h, the same sample without Ni^2+^ solution was applied as the reference. Their absorbances (*A*) of the complex were detected at the wavelength of 269 nm.

## 3. Results and discussions

### 3.1 Explorations of degradation conditions of ampicillin

**Fig. 1:**
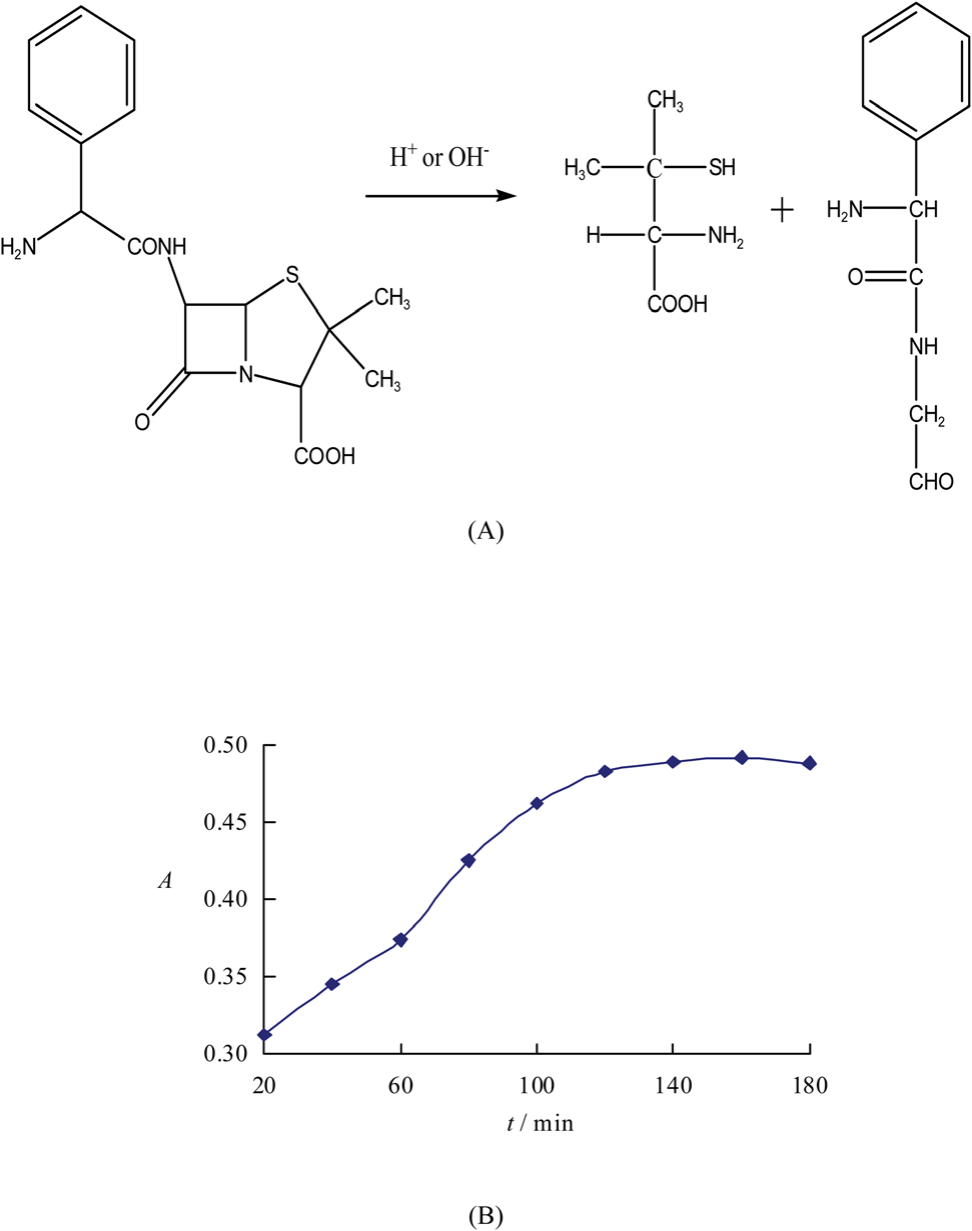
Degradation process (A) and degradation time (B) of ampicillin.

Ampicillin is difficult to be dissolved in the water but soluble in dilute alkali or dilute acid. However, it can be easily degraded into the penicillamine and penilloaldehyde with benzyl in dilute alkali or dilute acid, as shown in Fig. 1. Not only the hydroxyl group (O) of carboxyl, thiol group (S) and amino group (N) linking to the two adjacent carbons of penicillamine but also the amino group (N) linking to the two adjacent carbons of penilloaldehyde can combine with the adjacent atoms containing lone pair electrons to form the five-membered ring compounds by reacting with metals. Therefore, the content of ampicillin can be determined indirectly *via* the complex between ampicillin degradation products and metals. However, the peak intensity of acid degradation products of ampicillin is weaker than that of alkaline degradation products, so as to use the alkaline condition for degradation. 10 portions of equivalent degradation solution of ampicillin are prepared according to the method 2.2.1, and then heated for 20 ∼ 180 min respectively. It is found that the absorbance of alkaline degradation products of ampicillin increases along with the time, and the reaction tends to be more completed. The absorbance keeps stable when the reaction time shortens to or less than 120 min. So the optimized time for degradation is selected as 120 min.

### 3.2 Selection of metal ions

The metals including Cu^2+^, Co^2+^, Ni^2+^, Mn^2+^ and Zn^2+^ are combined with equivalent alkaline degradation products of ampicillin respectively. The studies find that new characteristic peaks are showed in the above complexes except for Zn^2+^. And the absorbance of the Ni^2+^-complex by alkaline degradation solution of ampicillin is the biggest. Therefore, Ni^2+^ is applied for the experiment.

### 3.3 Study on characteristic spectrums

**Fig. 2:**
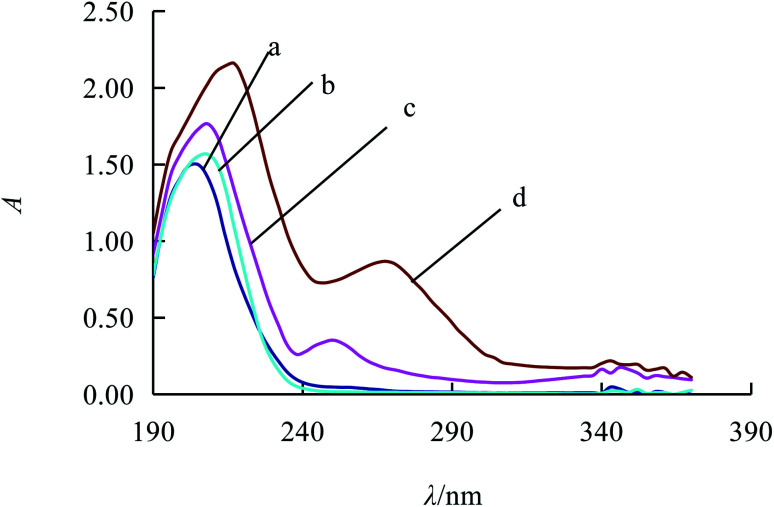
Absorption spectra of the different solutions (a 2.00 × 10^−4^ mol L^−1^ Ni^2+^, b 4.00 × 10^−4^ mol L^−1^ ampicillin, c 4.00 × 10^−4^ mol L^−1^ alkaline degradation products of ampicillin, d 4.00 × 10^−4^ mol L^−1^ complex of alkaline degradation products of ampicillin and Ni^2+^).

The distilled water is taken as the blank reference while UV is utilized to determine the Ni^2+^ liquid, ampicillin standard solution, alkaline degradation products of ampicillin and the complex respectively. According to Fig. 2, it is found that the absorption peaks of Ni^2+^ solution (curve *a*) and ampicillin solution (curve *b*) are respectively at 208 nm and 202 nm. At the same time, there are two characteristic absorption peaks of alkaline degradation products of ampicillin respectively at 208 nm and 250 nm (curve *c*) and another two characteristic absorption peaks of complex respectively at 217 nm and 269 nm (curve *d*). The absorption peak is selected as 269 nm due to its less interference than that around 217 nm.

**Fig. 3:**
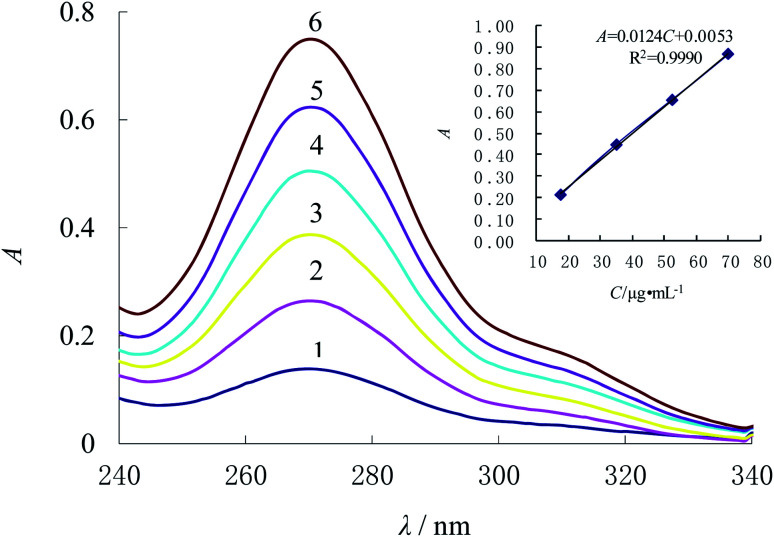
Absorption spectra of the complex for different concentrations of ampicillin with 0.20 mL 1% ammonium hydroxide and 2.00 × 10^−4^ mol L^−1^ Ni^2+^. From 1 to 6, ampicillin is 6.00 × 10^−5^ mol L^−1^, 1.20 × 10^−4^ mol L^−1^, 1.80 × 10^−4^ mol L^−1^, 2.40 × 10^−4^ mol L^−1^, 3.00 × 10^−4^ mol L^−1^, 3.60 × 10^−4^ mol L^−1^, respectively.

In this section, the concentrations of alkaline degradation products for ampicillin are changed. According to method 2.3, the determination result shown as Fig. 3 is that the absorbance increases along with the elevation of concentration (*C*) for the alkaline degradation solution of ampicillin, which presents a favorable linear relationship. In the range from 17.47 μg mL^−1^ to 69.88 μg mL^−1^, the regression equation is *A* = 0.0124*C* + 0.0053 with the *R*^2^ of 0.9990, and the detection limit is 0.52 μg mL^−1^ calculated by 3SD/*k*. The molar absorption coefficient (*ε*) was 4.28 × 10^3^ L mol^−1^ cm^−1^. Compared with other methods for ampicillin detection, the linear range of this method is better or the detection limit is lower, as shown in Table 1.

**Table 1:** The comparison of other methods for determination of ampicillin

Method	Linear range (μg mL^−1^)	Detection limit (μg mL^−1^)	Ref.
Spectrophotometric methods	5–30	0.162	7
Chromogentic spectrum	2.0–80	1.5	13
Charge-transfer spectrophotometry	0–40	—	23
High performance liquid chromatography	5.32–31.92	—	24
Indirect UV-Vis	17.47–69.88	0.52	This work

### 3.4 Study on the reaction time and temperature

**Fig. 4:**
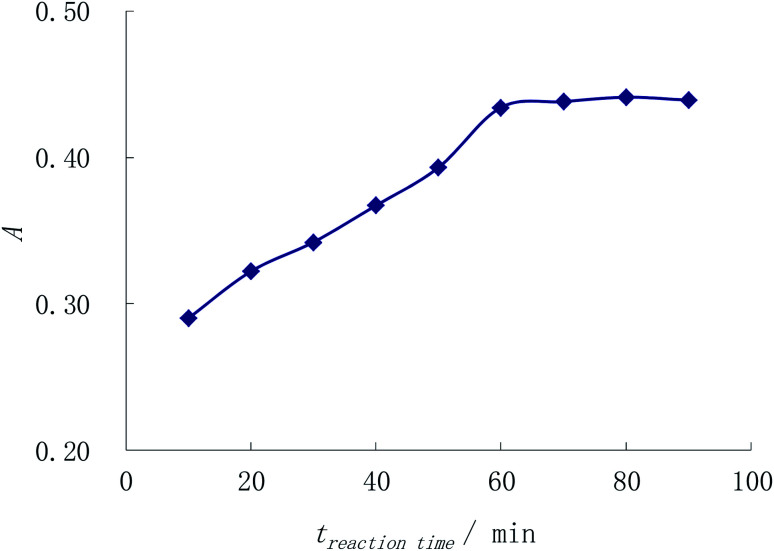
Selection for the reaction time of the complex.

Immobilizing other conditions, the complexing reaction time is observed by method 2.3. Fig. 4 shows that the absorbance enhances with the reaction time while the absorbance keeps the maximum and unchanged when the time lengthened to or more than 60 min. So the reaction time is chosen as 1 h.

**Fig. 5:**
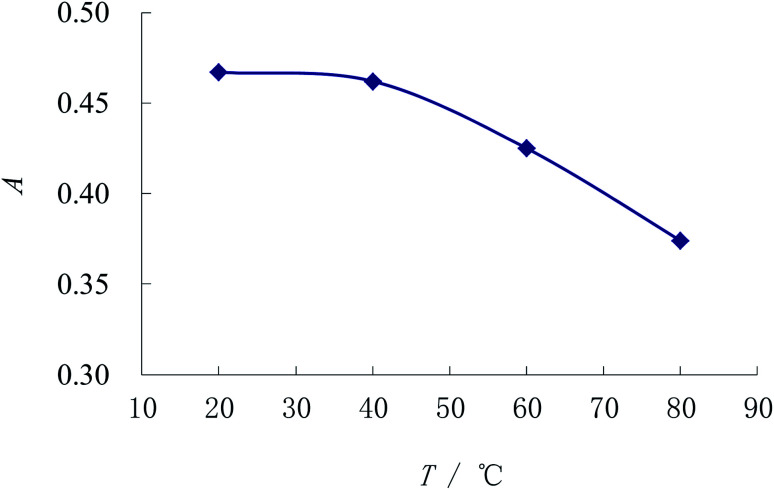
Selection for the reaction temperature of the complex.

The method 2.3 is also applied to investigate the complexing temperatures effect. Fig. 5 indicates that the absorbance is the best and the most stable when the temperature is below 40 °C, after which the absorbance of the complex begins to decrease. As a result, the room temperature is adopted for the experiment.

### 3.5 Optimization of pH

**Fig. 6:**
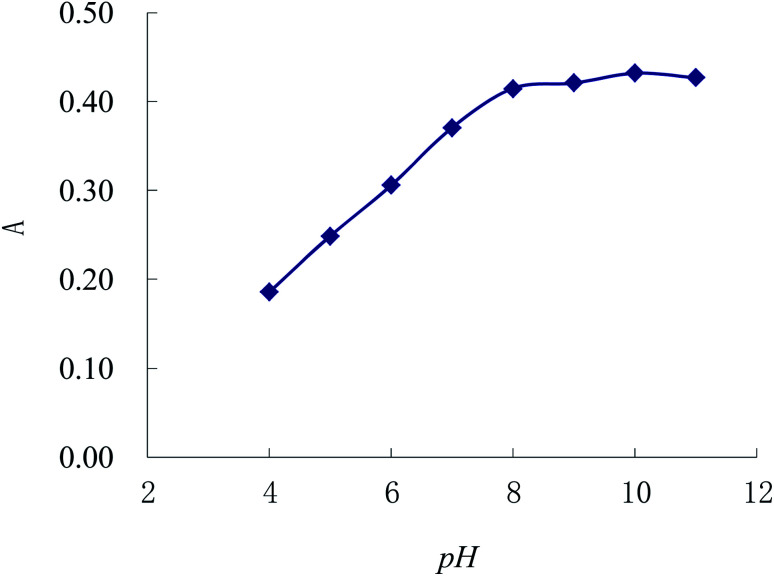
Selection for the pH of the complex.

In this section, we use NaOH and HCl solutions to adjust the pH. According to method 2.3, Fig. 6 shows that the absorbance increases along with the elevation of pH, and the absorbance tends to be the maximum and stable when the pH is over 8.00. In addition, the absorbance of complex within the ammonium hydroxide is more sensitive than that in NaOH solution. Under the fixed conditions but modifying the dosage of 1% ammonium hydroxide, the absorbance enhances with the increase of the dosage of ammonium hydroxide until the volume of ammonium hydroxide improves to 0.20 mL; but the absorbance declines when the volume of ammonium hydroxide goes beyond 0.20 mL. Therefore, 0.20 mL of 1% ammonium hydroxide is applied for regulating the pH value in the experiment.

### 3.6 Effects of adding order of reagents

According to method 2.3, three groups of reagents in different orders are added as follows, including ① alkaline degradation solution of ampicillin, 1% ammonium hydroxide and Ni^2+^ solution; ② alkaline degradation solution of ampicillin, Ni^2+^ solution and 1% ammonium hydroxide; ③ Ni^2+^ solution, 1% ammonium hydroxide and alkaline degradation solution of ampicillin; respectively. The absorbance of complex above is determined to be from the large to small as ① > ② > ③. As a result, the first group is adopted as the adding order for the experiment.

### 3.7 Discussions on stability constant and reaction mechanism of complex

**Fig. 7:**
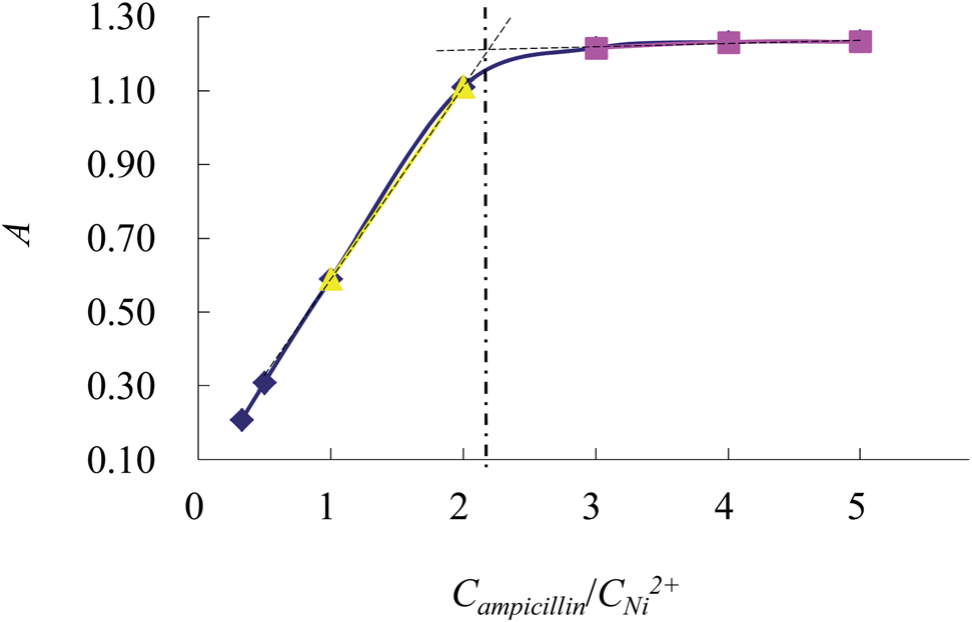
Complex stoichiometry.

As shown in Fig. 7, *A*_1_ is the absorbance of the complex when the *C*_ampicillin_/*C*_Ni_^2+^ is 2 : 1. *A*_max_ is the maximum absorbance of the complex. *A*_1_ is lower than *A*_max_, which suggests that the partial complexes are dissociated. The dissociation degree (*α*) and the stability constant (*K*) are defined as followings:
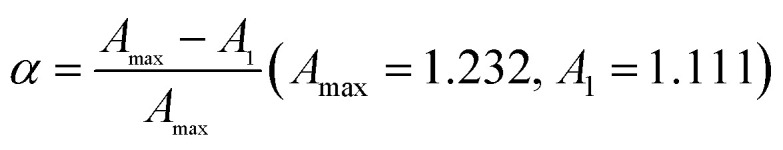

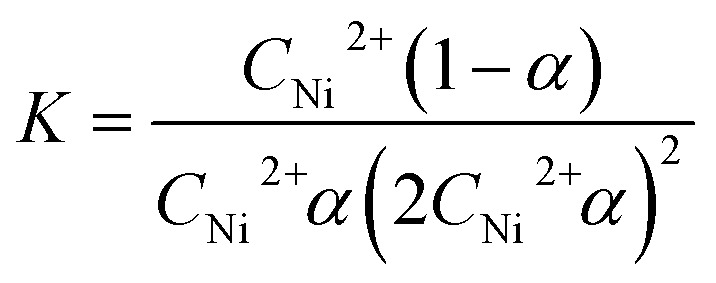


According to the equations, *α* is calculated as 0.0982 and *K* is 5.95 × 10^9^. The formed complex has a good stability.

Alkaline degradation solution of ampicillin and 1% ammonium hydroxide are added into one test tube in order. In another test tube, ampicillin alkaline degradation solution, 1% ammonium hydroxide and Ni^2+^ solution are added in sequence. Then 10 mL acetone is put into the two test tubes above mentioned and stirred well. After standing for 30 min, white alkaline degradation products of ampicillin and orange complex are separated out. Both the educt solids are washed with acetone for 3 times and then both are dried in the air naturally.

**Fig. 8:**
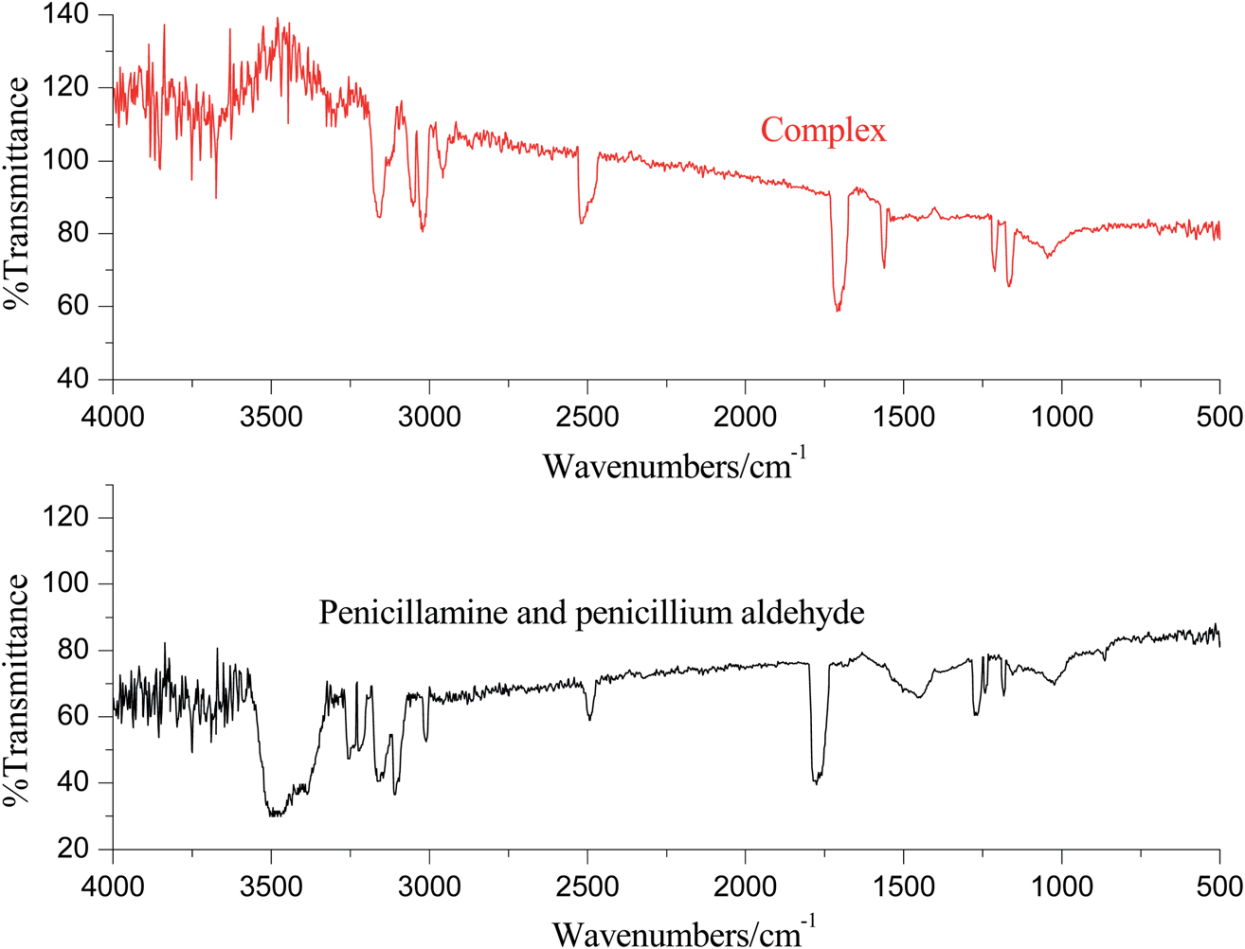
Infrared spectrum of the degradation products of penicillin and its complex.

**Fig. 9:**
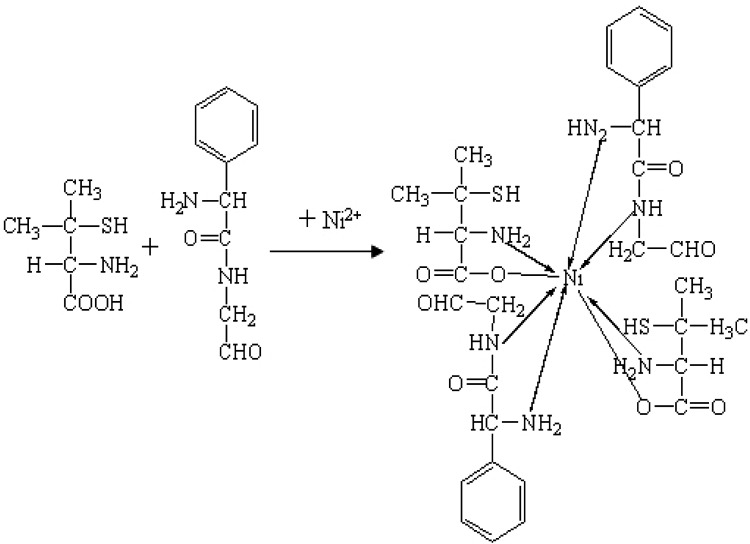
Reaction mechanism of the complex formation.

The IR spectra are displayed in Fig. 8. As the results shown, there is a strong stretching vibration of hydroxyl group of carboxyl of penicillamine in 3486 cm^−1^, but the absorption peak disappears in the complex.^25^ There is a stretching vibration of C=O of penicillamine in 1765 cm^−1^, and the absorption peak disappears in the complex. There are stretching vibrations of –COO– at 1716 cm^−1^ and 1553 cm^−1^ in the complex, which illustrates that Ni^2+^ replaces H of –COOH–. Moreover, there are absorption peaks of N–H in penicillamine at 3252 cm^−1^ and 3220 cm^−1^, while the absorption peaks of complex shifts to 3163 cm^−1^ and 3123 cm^−1^, which shows that atom N of penicillamine is involved in the coordination. The absorption peak of –SH keeps at the same location before and after the coordination reaction, which suggests that atom S isn't involved in the complex. There are two absorption peaks of N–H of primary amine in penilloaldehyde at 3155 cm^−1^ and 3099 cm^−1^ while the peaks shift to 3050 cm^−1^ and 3018 cm^−1^ in the complex. It reveals that Ni^2+^ and primary amine of penilloaldehyde are in coordination. Besides, there is a single absorption peak of N–H of secondary amine in penilloaldehyde at 3010 cm^−1^ while the peak shifts to 2953 cm^−1^ in the complex. It proves that the secondary amine of penilloaldehyde participates is in the coordination. To sum up, the reaction mechanism can be deduced as Fig. 9.

### 3.8 Interference experiment

2.00 mL alkaline degradation solution of ampicillin is transferred accurately. Glucose, dextrin, starch, stearic acid, lactose and CMC-Na are respectively added into the above solution. The conclusion with relative error less than ±5% is shown that the starch has more interference than other ingredients, but it can be removed *via* filtration.

## 4. Recovery experiment with additions

The shells of 20 ampicillin capsules are taken to mix evenly. The ampicillin powders equivalent to the labeled amount of 0.0873 g are weighed and dissolved in NaOH solution, and then heated in the boiling water bath for 2 h. Appropriate amounts of HCl are added to neutralize the pH of the solution. The solution is transferred and shaken evenly to a 250 mL volumetric flask after refrigeration, so as to obtain the filtrate for standby.

0.80 mL of the above filtrate is taken accurately, from which the content of ampicillin is determined according to the method 2.3 (Table 2). Besides, another 0.80 mL filtrate is applied for the recovery experiment as seen in Table 3.

**Table 2:** Determination results of sample contents (*n* = 3)

Sample	Label value (μg mL^−1^)	Measured value (μg mL^−1^)	Equivalent to labeled (%)	RSD (%)
1	27.95	28.93	103.5	0.8
2		29.09	104.1	1.2
3		29.17	104.4	1.4

**Table 3:** Determination results of sample recovery rates (*n* = 3)^a^

Sample	Measured value (μg mL^−1^)	Added (μg mL^−1^)	Measured value after addition (μg mL^−1^)	Recovery (%)	RSD (%)
1	28.93	13.98	43.12	101.5	0.9
		27.95	56.51	98.68	1.5
2	29.09	13.98	42.96	99.21	1.3
		27.95	57.07	100.1	0.7
3	29.17	13.98	43.52	102.7	1.7
		27.95	57.31	100.7	1.4

a Notes: Sample 1: ampicillin capsules (Hunan Anbang Pharmaceutical. Co., Ltd, 0.25 g per capsule). Sample 2: ampicillin capsules (rhe United Laboratories Co., Ltd, 0.25 g per capsule). Sample 3: ampicillin capsules (Zhuhai United Laboratories (Zhongshan) Co., Ltd, 0.25 g per capsule).

## 5. Conclusions

Under the alkaline condition, ampicillin can be degraded to penicillamine and penicillium aldehyde. In ammonium hydroxide, the degradation products can react with Ni^2+^ to form the 2:1 complex with maximum absorbance at the 269 nm. There is a favorable linear relationship between the absorption intensity of the complex and the addition amount of ampicillin. A new method has been initiated for indirect determination of ampicillin, which shows a low detection limit, good repeatability and high sensitivity.

## Conflicts of interest

There are no conflicts to declare.

## Supplementary Material
